# Tuning Coordination in s-Block Carbazol-9-yl Complexes

**DOI:** 10.1002/chem.201406490

**Published:** 2015-03-17

**Authors:** Fabrizio Ortu, Graeme J Moxey, Alexander J Blake, William Lewis, Deborah L Kays

**Affiliations:** [a] School of Chemistry, University of Nottingham, University Park Nottingham, NG7 2RD (UK), Fax: (+44) 115-9513555 E-mail: Deborah.Kays@nottingham.ac.uk; [b] School of Chemistry, University of Manchester Manchester M13 9PL (UK); [c] Research School of Chemistry, Australian National University Canberra, ACT 0200 (Australia)

**Keywords:** alkaline earth metals, ligand effects, magnesium, N ligands, potassium

## Abstract

1,3,6,8-Tetra-*tert*-butylcarbazol-9-yl and 1,8-diaryl-3,6-di(*tert*-butyl)carbazol-9-yl ligands have been utilized in the synthesis of potassium and magnesium complexes. The potassium complexes (1,3,6,8-*t*Bu_4_carb)K(THF)_4_ (**1**; carb=C_12_H_4_N), [(1,8-Xyl_2_-3,6-*t*Bu_2_carb)K(THF)]_2_ (**2**; Xyl=3,5-Me_2_C_6_H_3_) and (1,8-Mes_2_-3,6-*t*Bu_2_carb)K(THF)_2_ (**3**; Mes=2,4,6-Me_3_C_6_H_2_) were reacted with MgI_2_ to give the Hauser bases 1,3,6,8-*t*Bu_4_carbMgI(THF)_2_ (**4**) and 1,8-Ar_2_-3,6-*t*Bu_2_carbMgI(THF) (Ar=Xyl **5**, Ar=Mes **6**). Structural investigations of the potassium and magnesium derivatives highlight significant differences in the coordination motifs, which depend on the nature of the 1- and 8-substituents: 1,8-di(*tert*-butyl)-substituted ligands gave π-type compounds (**1** and **4**), in which the carbazolyl ligand acts as a multi-hapto donor, with the metal cations positioned below the coordination plane in a half-sandwich conformation, whereas the use of 1,8-diaryl substituted ligands gave σ-type complexes (**2** and **6**). Space-filling diagrams and percent buried volume calculations indicated that aryl-substituted carbazolyl ligands offer a steric cleft better suited to stabilization of low-coordinate magnesium complexes.

## Introduction

In the quest for new sterically demanding nitrogen-based donors, carbazol-9-yls substituted in the 1- and 8-positions have emerged as a very versatile class of ligand, finding use in main group and transition metal coordination chemistry. Recent reports have highlighted interesting properties with potential applications: A fluorescent Cu^2+^ carbazolyl complex, which is a potentially highly selective tool for cyanide detection,[1] a Cr^2+^ complex of a *C*_2_-symmetrical bis(oxazolinyl)carbazolyl ligand, which has been effective in the asymmetric catalysis of Nozaki–Hiyama allylation reactions,[2] and a Fe^2+^ carbazolyl complex reported by Niwa and Nakada in 2012, which has been employed as a catalyst for enantioselective asymmetric epoxidations.[3] Despite their great popularity, the use of carbazolyl ligands in the investigation of s-block coordination chemistry has been relatively limited, with only a handful of structurally authenticated alkali metal complexes;[4–12] crystallographically characterized alkaline earth–carbazolyl complexes are even rarer.[13–15] Theoretical investigations on model pyrrolyl complexes of the heavier alkaline earth metals indicate that the Group 2 cations should exhibit a greater preference for multi-hapto bonding than the Group 1 metals in complexes featuring five-membered amide rings.[14]

**Figure 1:**
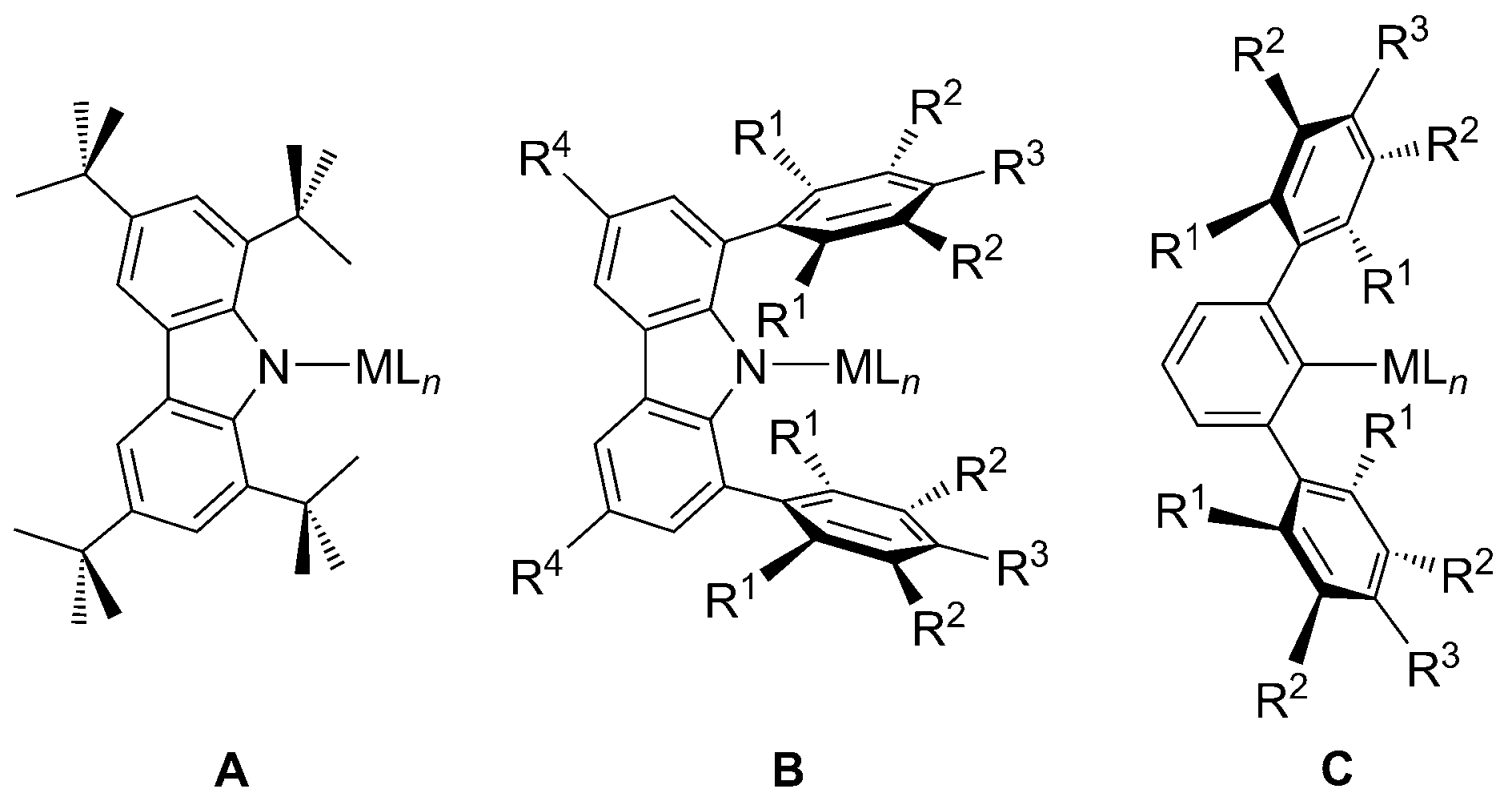
General schematic of 1,3,6,8-tetra-*tert*-butylcarbazol-9-yl (A), 1,8-diaryl-3,6-di(*tert*-butyl)carbazol-9-yl (B) and *m*-terphenyl (C) complexes.

Herein, we have focused our attention on two classes of 1,8-substituted carbazolyls as potential ligands for the Group 1 and 2 metals: 1,3,6,8-tetra-*tert*-butylcarbazolyl and 1,8-diarylcarbazolyl ligands (Figure 1 A and B). Due to their similarity, 1,8-diarylcarbazolyls have been proposed as competitors to *m*-terphenyl ligands (Figure 1 C).[9] The latter systems have been largely employed for the study of a range of unusual and highly reactive bonding modes for low-coordinate metal complexes, due to their great versatility and excellent steric hindrance.[16] It has been proposed that 1,8-diaryl substituted carbazolyl scaffolds could offer an even higher degree of protection around the steric pocket, compared to their *m*-terphenyl analogues.[9, 17] The ease of variation of the aryl substituents in the 1- and 8-positions of carbazoles and, consequently, the ability to tune their steric properties, makes them good candidates as proligands for the extremely challenging task of stabilizing low-coordinate metal complexes. Carbazolyl ligands are also preferable to *m*-terphenyls for stabilizing heavy alkaline earth (Ae) compounds, owing to the higher stability of complexes featuring Ae–N bonds compared to Ae–C interactions, particularly for the heavier congeners.[18]

**Figure 2:**
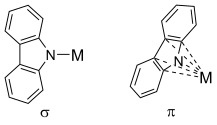
Schematic representation of mono-hapto σ-bonding and multi-hapto π-bonding modes of carbazolyl ligands.

Moreover, complexes of the 1,3,6,8-tetra-*tert*-butylcarbazol-9-yl ligand have been shown to display different coordination motifs from those featuring aryl-substituted carbazolyl ligands.[17, 19] The steric load near the amido nitrogen, guaranteed by the bulky *tert*-butyl groups in the 1- and 8-positions, reduces the likelihood of the cation forming σ complexes; instead, the metal is pushed below the plane of the carbazolyl ligand. Therefore, 1,3,6,8-tetra-*tert*-butylcarbazol-9-yl acts as a multi-hapto ligand, with a high tendency to form π complexes (Figure 2). However, upon substitution with aryl groups in the 1- and 8-positions, the ligand behaves as a classic amido σ donor (Figure 2). In a previous report, we have described the structural differences observed in a series of Group 1 complexes obtained with the 1,3,6,8-tetra-*tert*-butylcarbazol-9-yl ligand,[11] which arise from the increase in the size of the metal cations descending the Group. This work aims at further elucidating the different behaviour in terms of bonding modes and coordination motifs, expanding the study to 1- and 8-aryl-substituted carbazol-9-yl frameworks, extending them to the coordination chemistry of the Ae metals.

Herein, the use of potassium complexes of 1,3,6,8-tetra-*tert*-butylcarbazol-9-yl and 1,8-diaryl-3,6-di(*tert*-butyl)carbazol-9-yl ligands in metathetical reactions with MgI_2_ to form the corresponding Hauser bases is described. Structural investigations on the potassium and magnesium derivatives of these ligands highlight significant differences in the coordination motifs depending on the 1,8-substituents, with 1,8-di(*tert*-butyl) substituted ligands affording π-type compounds, in which the metal cations are positioned more below the coordination plane of the ligands and the use of 1,8-diaryl substituted ligands, which affords σ-type complexes.

## Results and Discussion

### Synthesis of potassium salts

**Scheme 1:**
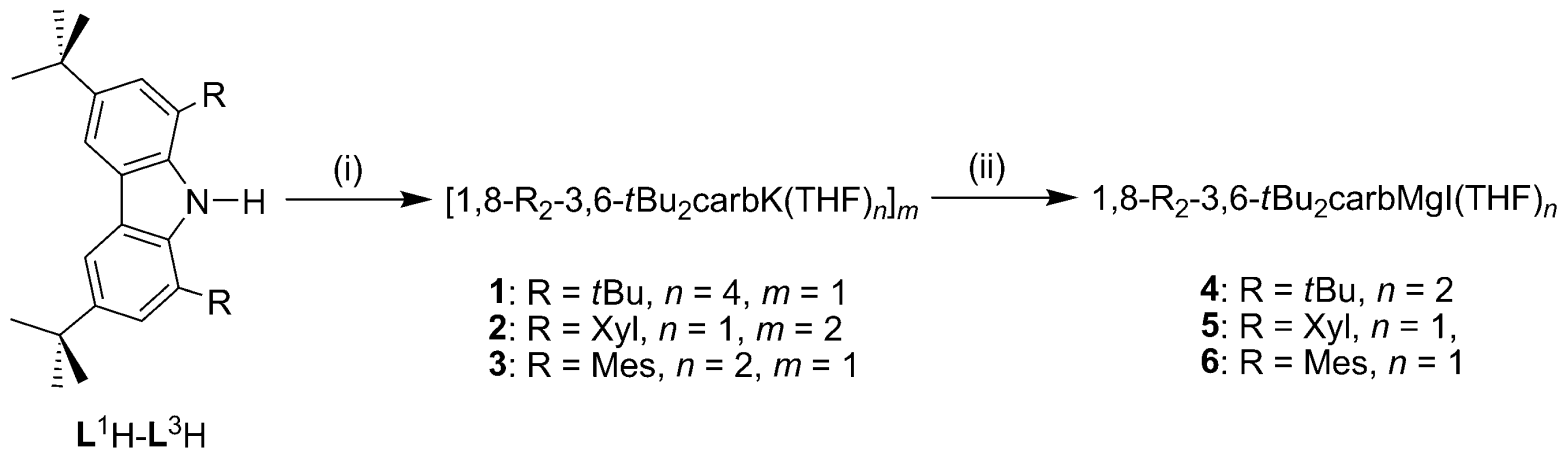
General reaction Scheme for the synthesis of potassium salts (1–3) and magnesium complexes (4–6). Reaction conditions: (i) 1:1.2 1,8-R_2_-3,6-*t*Bu_2_carbH:KH, THF, −78 °C to room temperature, 16 h, −H_2_; (ii) 1:1 K salt/MgI_2_, THF, −78 °C to room temperature, 16 h, −KI. The synthesis of complex 1 has been reported previously.[11]

Minor modification of a methodology developed by Gibson and co-workers, which allows the substitution of different aryl groups in the 1- and 8-positions of a carbazole ring, gave 1,8-Xyl_2_-3,6-*t*Bu_2_carbH (**L^2^**H) and 1,8-Mes_2_-3,6-*t*Bu_2_carbH (**L^3^**H),[20] thus facilitating convenient tuning of the steric properties of the ligand. The synthesis of the potassium salt (1,3,6,8-*t*Bu_4_carb)K(THF)_4_ (**1**) has been previously reported;[11] the same protocol was used with carbazoles **L^2^**H and **L^3^**H, affording the potassium salts [(1,8-Xyl_2_-3,6-*t*Bu_2_carb)K(THF)]_2_ (**2**) and (1,8-Mes_2_-3,6-*t*Bu_2_carb)K(THF)_2_ (**3**; Scheme 1). Pure samples of **2** and **3** have been isolated in good yields and characterized by spectroscopic and analytical techniques.

**Figure 3:**
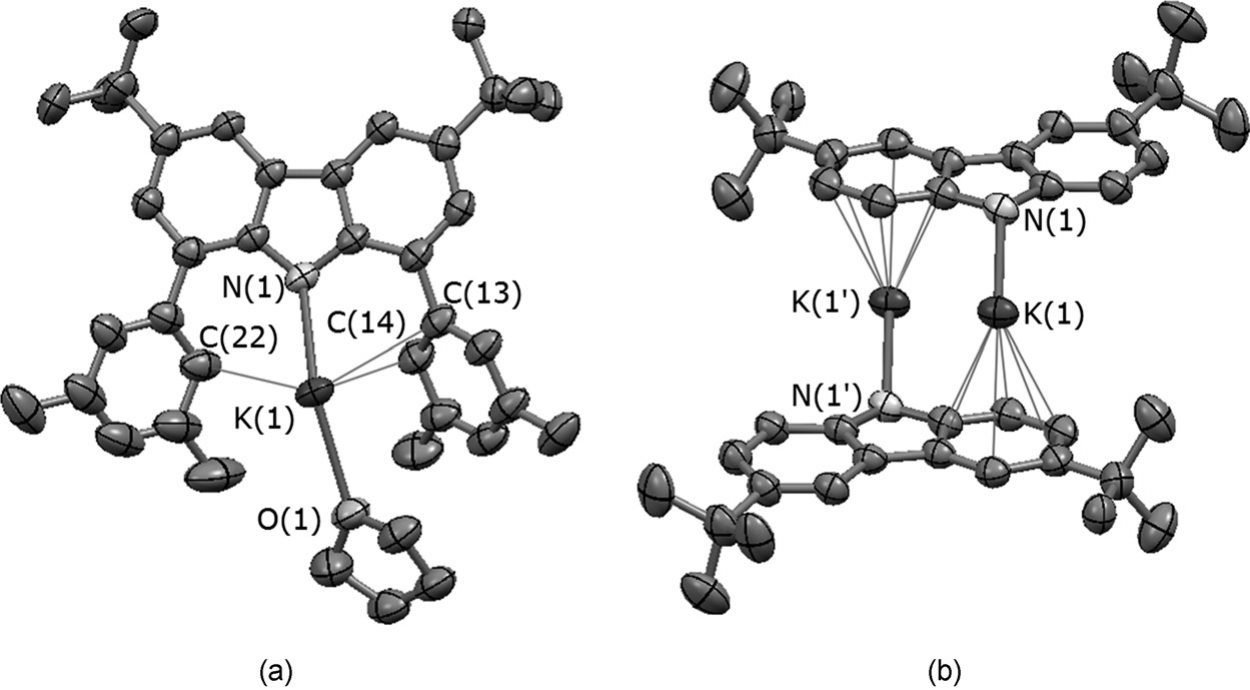
(a) Monomeric unit of 2 with displacement ellipsoids set at the 50 % probability level. Hydrogen atoms have been omitted for clarity. (b) Molecular structure of the dimer 2 with displacement ellipsoids set at 50 % probability level. Hydrogen atoms, aryl substituents and THF molecules have been omitted for clarity. Symmetry operation used for generating equivalent atoms: ′=1−*x*, 1−*y*, 1−*z*.

Crystals of **2** suitable for X-ray diffraction studies were grown from a concentrated hexane solution stored at −30 °C (Figure 3; relevant bond lengths and angles can be found in Table 1). This compound crystallizes as a dimer [(1,8-Xyl_2_-3,6-*t*Bu_2_carbK)(THF)]_2_, in which the K^+^ ions are bound to the pyrrolyl nitrogen of one carbazolyl unit with a short K(1)–N(1) bond length (2.758(3) Å), which is notably shorter than that for **1** (2.9053(19) Å)[11] and features η^6^-interactions with one of the condensed six-membered rings of the second ligand, displaying K**⋅⋅⋅**C distances ranging between 3.166(3)–3.265(3) Å. The coordination sphere of each potassium cation is saturated through the coordination of a THF molecule. Thus, it can be seen that the K^+^ ions can coordinate to both hard and soft Lewis bases within these dimeric structures. The distance between the centroid on the condensed five-membered ring and the cation is 2.9072(16) Å. The cations are further stabilized by long-range interactions with carbon atoms C(13) (K(1)**⋅⋅⋅**C(13) 3.277(4) Å), C(14) (K(1)**⋅⋅⋅**C(14) 3.235(4) Å) and C(22) (K(1)**⋅⋅⋅**C(22) 3.386(4) Å) on the flanking xylyl rings. Similar interactions between the potassium cations and flanking phenyl substituents were observed in [1,8-Ph_2_-3,6-Me_2_carbK]_2_.[9] Similar to the 1,8-diphenylcarbazolyl dimer reported by Aldridge and co-workers, there appears to be some degree of conformational flexibility inherent within the xylyl-substituted carbazolyl ligand in **2**, allowing the formation of close secondary inter- and intramolecular metal–arene interactions.

**Table 1:** Selected bond lengths [Å] and angles [°] for 2 (M=K) and 4, 6 a and b (M=Mg)

	2	4	6 a	6 b
M–N	2.758(3)	2.101(5)	2.009(6)	1.979(11)
M–C(1)		2.850(5)		
M–C(12)		2.824(6)		
M–O	2.735(3)	2.047(4) 2.040(4)	1.967(6)	1.970(11)
M–I		2.7126(19)	2.649(3)	2.646(10)
M–C(carb-η^6^)	2.9072(16)			
M–C(Ar)	3.277(4) [C(13)]		2.742(8) [C(22)]	
	3.235(4) [C(14)]		3.606(8) [C(13)]	
	3.386(4) [C(22)]			
N-M-O		109.49(19) 110.80(19)	118.3(3)	126.4(6)
N-M-I		131.20(16)	127.6(2)	128.2(5)
O-M-O		97.55(19)		
O-M-I		100.09(13) 102.52(13)	107.63(18)	105.0(4)
				
M-N-C-C	148.3(2)	103.6(4)	165.2(4)	160.4(6)

**Figure 4:**
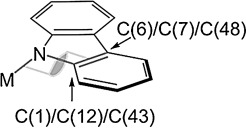
Deviation of the Mg from planar coordination and dihedral Mg-N-C-C angle.

For the series of Group 1 complexes of the ligand **L**^1^, an increase in the hapticity of the ligand–metal binding has been observed as the Group is descended from Li to Na to K, the larger cation sizes also being concomitant with increase in coordination of THF molecules.[11] In compound **1**, the carbazolyl ligand binds the metal cation in a π fashion with the five-membered condensed ring (dihedral angles calculated between the five-membered condensed ring and the metal centre gave a good indication of its position with respect to the coordination plane (Figure 4); K(1)-N(1)-C(1)-C(6) 80.6(2)°), which contrasts with the near-linear coordination of K^+^ by the carbazolyl ligand in **2** (in which K(1)-N(1)-C(12)-C(7) 148.3(2)°), the latter being also similar to the value of 141.6(5)° observed in the dimer [1,8-Ph_2_-3,6-Me_2_carbK]_2_.[9] The driving force responsible for the formation of Aldridge and co-workers’ potassium dimer arises from the interaction between the lone pair of the carbazolyl–nitrogen and the metal cation of the second unit. Such interactions were not observed for **2**, in which, in the solid state, the aggregation of the two monomeric units is supported by an η^6^-interaction.

There are few structurally authenticated examples of a potassium cation η^6^-bound to a carbazolyl arene ring, and in all previous cases the K^+^ ion is part of a heterobimetallic structure.[21, 22] Of particular interest is [K(THF)][ZrCl_2_(NC_12_H_8_)_3_(THF)], in which: 1) the average length of the K**⋅⋅⋅**C interactions is 3.248(3) Å, a value consistent with the distances measured for **2** (ave. K**⋅⋅⋅**C 3.226(7) Å); 2) the complex features an additional η^2^-interaction with a second carbazolyl unit, with distances of 3.147(3) and 3.268(2) Å.[21] In previous literature examples, increasing alkali metal ionic radius tends to favour the adoption of a multi-hapto binding motif through the five-membered condensed ring of the carbazolyl ligand.[5, 7, 11] The behaviour of the K^+^ ion within dimer **2** appears to be closer to that in the powder X-ray diffraction structure of the unsolvated caesium carbazolyl complex Cs(NC_12_H_8_), which in the main displays a combination of η^1^-nitrogen and η^6^-arene interactions in the solid state.[8]

### Synthesis of Hauser bases

Potassium complexes **1**–**3** were employed for metathetical reactions with MgI_2_ in a 1:1 ratio according to Scheme 1. Following this synthetic route, compounds 1,3,6,8-*t*Bu_4_carbMgI(THF)_2_ (**4**), 1,8-Xyl_2_-3,6-*t*Bu_2_carbMgI(THF) (**5**) and 1,8-Mes_2_-3,6-*t*Bu_2_-carbMgI(THF) (**6**) were isolated and characterized. These complexes are highly sensitive to air and moisture, but are stable if stored under an inert atmosphere. Because of the high sensitivity of these Hauser bases, hydrolysis easily occurs during work-up, leading to the isolation of free amine, which was observed by ^1^H NMR spectroscopy and X-ray crystallography; such ease of decomposition significantly affected the overall yields. In particular, the isolation of the highly sensitive compound **4** proved to be a very challenging task, because the complex decomposes during manipulation of solids and solutions of this complex. However, we were able to thoroughly characterize complex **4** by ^1^H and ^13^C{^1^H} NMR and IR spectroscopy techniques. The ^1^H NMR analysis was particularly indicative of the instability of the complex: the [D_6_]benzene solution turned blue in a few hours, and a significant broadening of the spectral lines was observed, which is associated with the formation of a quantity of the previously observed radical;[23] additional signals for the free amine were also detected. Due to the high sensitivity of the compound, a satisfactory elemental analysis could not be obtained. Complexes **5** and **6** have been characterized by spectroscopic methods, and the elemental microanalyses of these compounds are all in good agreement with the formation of heteroleptic species of the general formula LMgI(THF)_*n*_ (*n*=0, 1).

**Figure 5:**
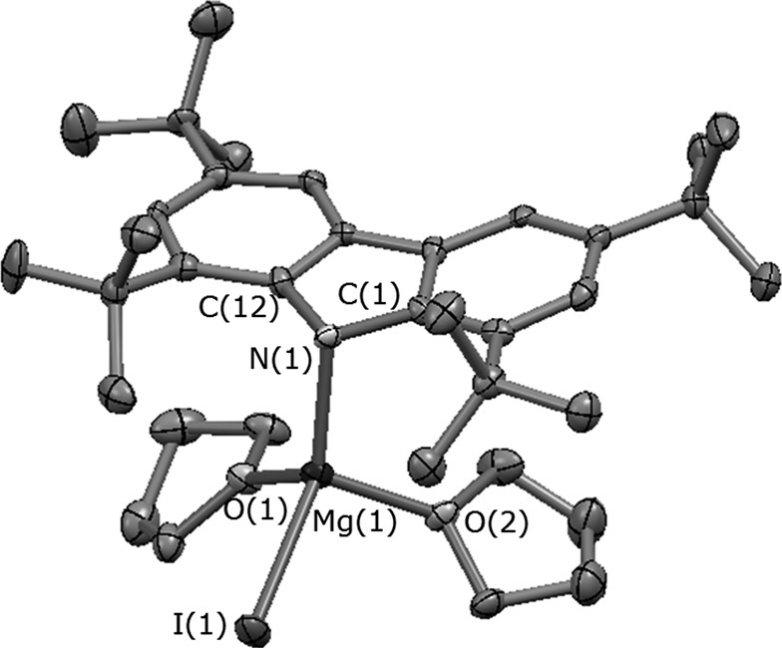
Molecular structure of 4 with displacement ellipsoids set at the 50 % probability level. Hydrogen atoms have been omitted for clarity.

Single crystals of **4** of suitable quality for X-ray diffraction studies were obtained from a concentrated solution of the complex in hexane at room temperature (Figure 5; relevant bond lengths and angles can be found in Table 1). In the solid state, the metal is coordinated by the carbazolyl ligand via the amido nitrogen (Mg(1)–N(1) 2.101(5) Å); the metal achieves saturation of the coordination sphere by binding one iodide (Mg(1)–I(1) 2.7125(19) Å) and two THF molecules (Mg(1)–O(1) 2.047(4) Å, Mg(1)–O(2) 2.040(4) Å), displaying a distorted tetrahedral geometry (N(1)-Mg(1)-O(1) 110.80(19)°, O(1)-Mg(1)-O(2) 97.55(19)°, N(1)-Mg(1)-O(2) 109.49(19)°, N(1)-Mg(1)-I(1) 131.20(16)°, O(1)-Mg(1)-I(1) 100.09(13)°, O(2)-Mg(1)-I(1) 102.52(13)°). The four-coordinate magnesium cation is positioned below the ligand plane (Mg(1)⋅⋅⋅carb_*centr*_ 2.828(2) Å; Mg(1)-N(1)-C(1)-C(6) 103.7(3)°), with Mg**⋅⋅⋅**C_α_ distances (Mg(1)–C(1) 2.850(5) Å, Mg(1)–C(12) 2.824(6) Å), which are shorter than the sum of their van der Waals radii (*r*_sum_=3.90–4.15 Å);[24] thus, it is tentatively concluded that the coordination of the metal centre is supported by a pseudo-π interaction. This coordination is similar to that for the analogous ethyl complex 1,3,6,8-*t*Bu_4_carbMg(Et)(THF)_2_ (in which Mg–N 2.087(3) Å, Mg-N-centroid 117.4°).[13]

**Figure 6:**
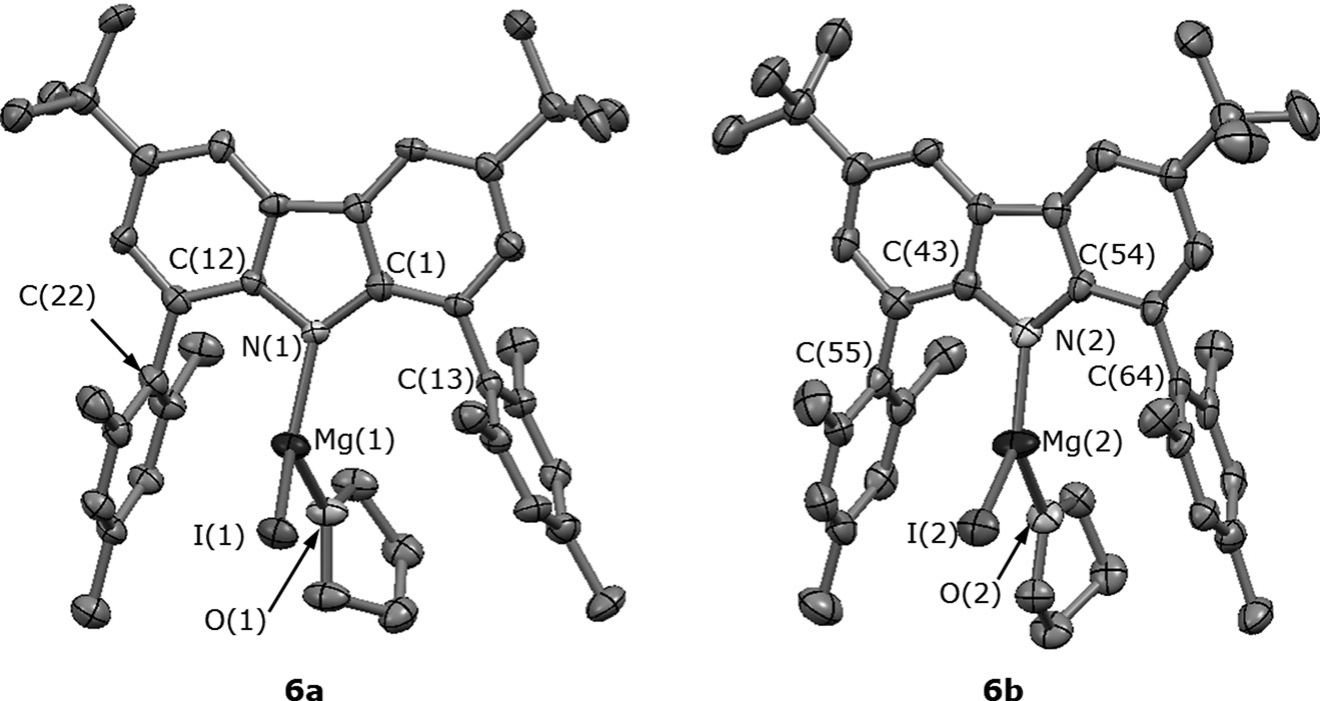
Molecular structure of the two crystallographically independent molecules of 6 [6 a (left) and 6 b (right)] with displacement ellipsoids set at the 50 % probability level. Hydrogen atoms have been omitted for clarity. The view is not representative of the relative orientation of the two molecules 6 a and b in the asymmetric unit, but has been chosen for clarity.

An X-ray study was performed on single crystals grown from a concentrated solution of **6** in hexane at −30 °C. The crystal structure of this complex is shown in Figure 6. The asymmetric unit includes two crystallographically independent LMgI(THF) units (**6 a** and **b**), which display similar connectivity. The magnesium centres are formally three-coordinate, which is a very rare coordination environment for such complexes;[25, 26] compound **6 a** also exhibits a long-range interaction between the mesityl C(22) and the metal cation. Carbazolyl ligands substituted with aryl groups in the 1- and 8-positions have been shown to offer a higher degree of steric protection compared to their *m*-terphenyl analogues.[9] This seems to be the case here, because **6** features a formally three-coordinate Mg^2+^ in the solid state, whereas the terphenyl Grignard reagents (which exhibit a range of aryl substituent sizes: Ph, *p*-Tol (4-MeC_6_H_4_), Mes, Trip (2,4,6-*i*Pr_3_C_6_H_2_)) show four-coordinate Mg^2+^ or greater.[27]

The connectivity within **6** differs significantly from that for **4**, resembling the behaviour of a more classic amido σ-type complex. Furthermore, in compound **4**, there is significant puckering of the carbazolyl ring (angle between the best mean planes of the condensed phenyl rings for **4** is 10.1°) compared to a relatively planar ligand in **6**, due to the more sterically demanding 1,3,6,8-tetra-*tert*-butylcarbazolyl ligand, although it is also acknowledged that other factors, such as coordination number, will also contribute to the steric strain around the metal centre. The proximity of the *tert*-butyl substituents to the nitrogen donor in **1** and **4** is responsible for the positioning of the metal centre below the coordination plane in a half-sandwich conformation (see Figure S2 in the Supporting Information). Conversely, in compounds **2** and **6**, the proximity of the relatively flat flanking aryl rings can create a steric pocket to accommodate the Mg^2+^ cation in a position closer to a coplanar arrangement with respect to the carbazolyl plane, in which, in the case of **6**, the metal nestles comfortably within the cleft created by the two flanking mesityl substituents (see Figure S2 in the Supporting Information). Certainly, the flat aryl substituents seem to create a near perfect steric pocket to protect the magnesium in **6**, which is precluded in **4** by the *tert*-butyl groups, which have a larger footprint in three dimensions.

In an attempt to understand further the steric demands of the *tert*-butyl- versus aryl-substituted carbazolyl ligands in these complexes, we also embarked on percent buried volume calculations (%*V*_Bur_), which have been utilized to determine the steric bulk of N-heterocyclic carbene (NHC) ligands.[28] For the Hauser bases, the value of %*V*_Bur_ is 51.9 % for **4** and 54.7 % for **6**. By this measure, the greater steric protection will originate from the 1,8-Mes_2_-3,6-*t*Bu_2_carb^−^ ligand, and presumably results from the differing coordination mode due to the more disc-shaped mesityl groups; additionally, in compound **4**, the lower value of %*V*_Bur_ could arise from the distortion of the carbazolyl plane. The percent buried volume in the potassium compound of the 1,3,6,8-*t*Bu_4_carb^−^ ligand (for **1** %*V*_Bur_ 56.9 %) is somewhat higher than in **4**, presumably because the carbazolyl ligand is more planar in the former complex. In the case of the potassium complexes, the aryl-substituted carbazolyl ligands contribute to a lower percent buried volume, with a value of %*V*_Bur_ 54.4 % for **2**.

### Conclusion

A series of substituted carbazol-9-yl ligands has been used in the synthesis of potassium salts, which have been further employed as ligand-transfer reagents for the synthesis of heteroleptic magnesium halide complexes. Sterically demanding carbazolyl ligands can stabilize rare examples of three-coordinate alkaline earth Grignard analogues. X-ray diffraction studies on the potassium and magnesium derivatives highlight significant differences in the coordination motifs depending on the substituents on the 1- and 8-positions: 1) 1,8-di(*tert*-butyl) substituted ligands afford π-type compounds (**1** and **4**), in which the carbazolyl ligand acts as a multi-hapto donor, with the metal cations positioned below the coordination plane in a half-sandwich conformation; and 2) the use of 1,8-diaryl substituted ligands gave σ-type complexes (**2** and **6**). Space-filling diagrams and percent buried volume calculations indicate that aryl-substituted carbazolyl ligands offer a steric cleft better suited to stabilization of low-coordinate magnesium complexes. These investigations indicate that by choice of suitable carbazolyl ligands, it may be possible to tailor the coordination environment of the resulting amide complexes to suit particular targets.

## Experimental Section

### General remarks

**Figure 7:**
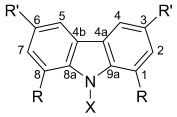
General numbering scheme of carbazol-9-yl compounds.

All of the potassium and magnesium compounds prepared herein are air and moisture sensitive; therefore, all reactions and manipulations were performed by using standard Schlenk line and glovebox equipment under an atmosphere of purified argon or dinitrogen. The hexane used was isohexane purchased from Fisher Scientific (contains <5% *n*-hexane); it was dried by passing through a column of activated 4 Å molecular sieves. THF was pre-dried over Na wire and freshly distilled over sodium benzophenone ketyl under nitrogen. All solvents were degassed in vacuo and stored over a potassium mirror (isohexane) or activated 4 Å molecular sieves (THF) prior to use. [D_6_]Benzene (Goss) was dried over potassium, and [D_8_]THF (Goss) was dried over CaH_2_. Both were degassed with three freeze/pump/thaw cycles prior to use. ^1^H and ^13^C{^1^H} NMR spectra were collected on Bruker AV 400, DPX 400 or DPX 300 spectrometers. Chemical shifts are quoted in ppm relative to TMS. IR samples were prepared as a Nujol mull between two KBr discs; the preparation of the sample was carried out in the glovebox under a nitrogen atmosphere. IR absorption spectra were recorded on a Bruker Tensor 27 FTIR spectrometer over a frequency range of 500–4000 cm^−1^. Elemental analyses were performed by Mr. Stephen Boyer at London Metropolitan University. Despite repeated attempts, a satisfactory elemental analysis for **4** could not be obtained, due to its very high sensitivity. This is a well-established issue for s-block organometallic complexes.[29] Ligand precursors **L^1^**H–**L^3^**H were synthesized by minor modifications of previous reported synthetic procedures;[9, 20, 30] details of the synthesis of **L^2^**H and **L^3^**H can be found in the Supporting Information. KH (Alfa Aesar) was purchased as a suspension in mineral oil; this was washed three times with isohexane and then dried in vacuo for 48 h. Anhydrous MgI_2_ (Sigma–Aldrich) was heated to 300 °C in vacuo overnight and stored under purified nitrogen. All other reagents were obtained from commercial sources and used as received. Yields refer to purified products and are not optimized. The general numbering Scheme for the spectroscopic assignments for carbazolyl compounds is given in Figure 7.

### Synthesis of potassium salts

The synthesis of **1** has been reported previously.[11] The following general synthetic procedure was employed for the synthesis of the potassium carbazolyl complexes **2** and **3**. A solution of the carbazole in THF (20 cm^3^) was added dropwise to a suspension of KH in THF (10 cm^3^) at −78 °C, with a molar ratio carbazole/KH of approximately 1:1.2; the reaction was warmed slowly to room temperature and stirred overnight. The mixture was filtered and dried; the solid residue was washed with isohexane (2×10 cm^3^) and dried in vacuo, giving **2** and **3** as yellow green powders.

### [(1,8-Xyl_2_-3,6-*t*Bu_2_carb)K(THF)]_2_ (2)

From **L^2^**H (0.60 g, 1.2 mmol) and KH (0.06 g, 1.4 mmol); **2** (0.39 g, 0.7 mmol, yield 54 %). Crystals of **2** were grown from a concentrated isohexane solution at −30 °C. Spectroscopic data were obtained on an unsolvated sample. ^1^H NMR ([D_6_]benzene/[D_8_]THF, 298 K, 300.13 MHz): *δ*=1.60 (s, 36 H, C(C*H*_3_)_3_), 2.29 (s, 24 H, Ar-C*H*_3_), 6.75 (s, 4 H, Ar-C*H*), 7.71 (d, ^4^*J*_HH_=2.0 Hz, 4 H, carb-C*H*^2, 7^), 8.09 (s, 8 H, Ar-C*H*), 8.46 ppm (d, ^4^*J*_HH_=2.0 Hz, 4 H, carb-C*H*^4, 5^); ^13^C{^1^H} NMR ([D_6_]benzene/[D_8_]THF, 298 K, 75.47 MHz): *δ*=21.4 (Ar-*C*H_3_), 32.6 (C(*C*H_3_)_3_), 34.5 (*C*(CH_3_)_3_), 115.4 (carb-*C*H^4, 5^), 120.1 (carb-*C*H^2, 7^), 125.4 (carb-*C*^8a, 9a^), 126.4 (carb-*C*^4a, 4b^), 126.5 (Ar-*C*H), 127.5 (Ar-*C*H), 134.8 (carb-*C*^1, 8^), 136.6 (Ar-*C*), 144.6 (Ar-*C*(CH_3_)), 150.0 ppm (carb-*C*^3, 6^); IR (Nujol): 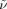
=3011 (w), 2936 (s), 1594 (s), 1407 (w), 1361 (w), 1290 (s), 1258 (w), 1226 (m), 1199 (w), 1170 (w), 1036 (m), 921 (w), 868 (m), 849 (s), 799 (w), 775 (w), 707 (m), 665 (w), 650 cm^−1^ (w); elemental analysis calcd (%) for C_80_H_96_K_2_N_2_O_2_ (1195.83): C 78.87, H 8.42, N 2.09; found: C 78.90, H 8.32, N 2.17.

### (1,8-Mes_2_-3,6-*t*Bu_2_carb)K(THF)_2_ (3)

From **L^3^**H (0.45 g, 0.9 mmol) and KH (0.04 g, 1.0 mmol); **3** (0.39 g, 0.6 mmol, yield 64 %). ^1^H NMR ([D_6_]benzene, 298 K, 400.07 MHz): *δ*=1.39 (m, 8 H, THF-C*H*_2_), 1.67 (s, 18 H, C(C*H*_3_)_3_), 1.99 (s, 6 H, Ar-C*H*_3_), 2.09 (s, 12 H, Ar-C*H*_3_), 3.50 (m, 8 H, THF-OC*H*_2_), 6.55 (s, 4 H, Ar-C*H*), 7.34 (d, ^4^*J*_HH_=2.0 Hz, 2 H, carb-C*H*^2, 7^), 8.63 ppm (d, ^4^*J*_HH_=1.9 Hz, 2 H, carb-C*H*^4, 5^); ^13^C{^1^H} NMR ([D_6_]benzene, 298 K, 100.63 MHz): *δ*=20.2 (Ar-*C*H_3_), 20.6 (Ar-*C*H_3_), 24.5 (THF-*C*H_2_), 32.8 (C(*C*H_3_)_3_), 34.7 (*C*(CH_3_)_3_), 68.6 (THF-O*C*H_2_), 115.4 (carb-*C*H^4, 5^), 119.9 (carb-*C*H^2, 7^), 124.2 (carb-*C*^8a, 9a^), 126.0 (carb-*C*^4a, 4b^), 127.6 (Ar-*C*H), 134.3 (Ar-*C*(CH_3_)), 135.3 (carb-*C*^1, 8^), 138.2 (Ar-*C*(CH_3_)), 142.9 (Ar-*C*), 150.3 ppm (carb-*C*^3, 6^); IR (Nujol): $\tilde \nu $
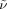
=3022 (w), 2957 (s), 1737 (w), 1609 (w), 1566 (w), 1416 (w), 1309 (w), 1294 (w), 1279 (s), 1245 (s), 1204 (m), 1184 (w), 1052 (s), 907 (m), 863 (w), 853 (s), 800 (w), 777 (w), 768 (w), 667 (w), 649 (w), 635 (w), 555 (w), 502 cm^−1^ (w); elemental analysis calcd (%) for C_46_H_60_KNO_2_ (698.07): C 79.15, H 8.66, N 2.01; found (%) C 78.88, H 8.55, N 2.10.

### Synthesis of 1,3,6,8-*t*Bu_4_carbMgI(THF)_2_ (4)

A solution of **1** (0.45 g, 1.1 mmol) in THF (20 cm^3^) was added dropwise to a suspension of MgI_2_ (0.33 g, 1.2 mmol) in THF (10 cm^3^) at −78 °C; the reaction was warmed slowly to room temperature and stirred overnight. Precipitation occurred, and volatiles were removed in vacuo. The solid residue was extracted with isohexane (10 cm^3^) and stored at −30 °C. Green crystals of **4** (0.02 g, 0.2 mmol, yield 21 %) were isolated from the isohexane solution. ^1^H NMR ([D_6_]benzene, 298 K, 400.07 MHz): *δ*=1.36 (br, 8 H, THF-C*H*_2_), 1.50 (s, 36 H, C(C*H*_3_)_3_), 3.77 (br, 8 H, THF-OC*H*_2_), 7.61 (br, 2 H, carb-C*H*^2, 7^), 8.27 ppm (d, 2 H, carb-CH^4, 5^); ^13^C{^1^H} NMR ([D_6_]benzene/[D_8_]THF, 298 K, 100.63 MHz): *δ*=25.1 (THF-*C*H_2_), 30.4 (3,6-*C*(CH_3_)_3_), 32.3 (1,8-*C*(CH_3_)_3_), 34.8 and 35.1 (1,8- and 3,6-*C*(CH_3_)_3_), 69.0 (THF-O*C*H_2_), 114.7 (carb-*C*H^2, 7^), 120.4 (carb-*C*H^4, 5^), 124.9 (carb-*C*^4a, 4b^), 132.0 (carb-*C*^1, 8^), 135.6 (carb-*C*^3, 6^), 142.3 ppm (carb-*C*^8a, 9a^); IR (Nujol): $\tilde \nu $
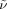
=3053 (w), 2970 (s), 1579 (m), 1493 (m), 1421 (w), 1292 (w), 1261 (s), 1215 (w), 1192 (m), 1096 (br s), 1023 (br s), 913 (m), 869 (s), 802 (s), 795 (w), 584 (m), 544 cm^−1^ (m).

### Synthesis of 1,8-Xyl_2_-3,6-*t*Bu_2_carbMgI(THF) (5)

A solution of **2** (0.35 g, 0.6 mmol) in THF (20 cm^3^) was added dropwise to a suspension of MgI_2_ (0.22 g, 0.8 mmol) in THF (10 cm^3^) at −78 °C; the reaction was warmed slowly to room temperature and stirred overnight. Precipitation occurred, and volatiles were removed in vacuo. The solid residue was washed with isohexane (10 cm^3^) and then extracted with THF (15 cm^3^). The yellow solution was concentrated to dryness in vacuo, and the residue was washed with isohexane (10 cm^3^), giving **5** as a white powder (0.07 g, 0.1 mmol, yield 17 %). ^1^H NMR ([D_6_]benzene/[D_8_]THF, 298 K, 400.07 MHz): *δ*=1.44 (s, 18 H, C(C*H*_3_)_3_), 1.49 (m, 4 H, THF-C*H*_2_), 2.22 (s, 12 H, C*H*_3_), 3.54 (m, 4 H, THF-OC*H*_2_), 6.83 (s, 2 H, Ar-C*H*), 7.31 (s, 4 H, Ar-C*H*), 7.61 (d, ^4^*J*_HH_=1.8 Hz, 2 H, carb-C*H*^2, 7^), 8.26 ppm (d, ^4^*J*_HH_=1.8 Hz, 2 H, carb-C*H*^4, 5^); ^13^C{^1^H} NMR ([D_6_]benzene/[D_8_]THF, 298 K, 100.63 MHz): *δ*=21.7 (Ar-*C*H_3_), 25.1 (THF-*C*H_2_), 32.5 (C(*C*H_3_)_3_), 35.3 (*C*(CH_3_)_3_), 67.3 (THF-O*C*H_2_), 116.2 (carb-*C*H^4, 5^), 124.3 (carb-*C*H^2, 7^), 125.2 (carb-*C*^4a, 4b^), 125.6 (carb-C^8a, 9a^), 126.8 (Ar-*C*H), 129.4 (Ar-*C*H), 137.1 (carb-*C*^1, 8^), 139.0 (Ar-*C*(CH_3_)_3_), 140.1 (Ar-*C*), 143.3 ppm (carb-*C*^3, 6^); IR (Nujol): $\tilde \nu $
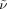
=2955 (s), 1599 (w), 1286 (w), 1261 (m), 1221 (w), 1095 (m), 1023 (s), 869 (w), 850 (w), 802 cm^−1^ (m); elemental analysis calcd (%) for C_40_H_48_IMgNO (710.02): C 67.66, H 6.81, N 1.97; found C 67.56, H 6.13, N 1.95.

### Synthesis of 1,8-Mes_2_-3,6-*t*Bu_2_carbMgI(THF) (6)

A solution of **3** (0.3 g, 0.4 mmol) in THF (20 cm^3^) was added dropwise to a suspension of MgI_2_ (0.14 g, 0.5 mmol) in THF (10 cm^3^) at −78 °C. Precipitation occurred, and volatiles were then removed in vacuo. The solid residue was extracted with isohexane (10 cm^3^), and crystals of **6** suitable for X-ray diffraction studies grew at −30 °C. The insoluble residue was extracted with THF (10 cm^3^), and the solvent was removed in vacuo, giving **6** as a white powder (0.09 g, 0.1 mmol, 31 %); ^1^H NMR ([D_6_]benzene, 298 K, 400.07 MHz): *δ*=1.42 (s, 18 H, C(C*H*_3_)_3_), 1.46 (m, 4 H, THF-C*H*_2_), 1.98 (s, 6 H, Ar-C*H*_3_), 2.09 (s, 12 H, Ar-C*H*_3_), 3.55 (m, 4 H, THF-OC*H*_2_), 6.73 (s, 4 H, Ar-C*H*), 7.26 (br, 2 H, carb-CH^2, 7^), 8.28 ppm (br, 2 H, carb-C*H*^4, 5^); ^13^C{^1^H} NMR ([D_6_]benzene, 298 K, 100.63 MHz): *δ*=20.1 (Ar-*C*H_3_), 20.6 (Ar-*C*H_3_), 24.4 (THF-*C*H_2_), 31.9 (C(*C*H_3_)_3_), 34.6 (*C*(CH_3_)_3_), 6.6 (THF-O*C*H_2_), 114.9 (carb-*C*H^4, 5^), 123.5 (carb-*C*H^2, 7^), 124.4 (carb-*C*^4a, 4b^), 124.7 (carb-*C*^8a, 9a^), 128.3 (Ar-*C*H), 135.5 (Ar-*C*), 136.3 (carb-*C*^1, 8^), 136.7 (Ar-*C*(CH_3_)), 137.1 ppm (Ar-*C*(CH_3_)), 142.2 (carb-*C*^3, 6^); IR (Nujol): $\tilde \nu $
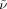
=2962 (s), 1610 (w), 1280 (m), 1242 (m), 1091 (m), 1041 (s), 871 (m), 851 (w), 762 (w), 675 (w), 652 (w), 626 (w), 558 (w), 509 cm^−1^ (w). Elemental microanalysis (measured on an unsolvated sample) calcd (%) for C_38_H_44_IMgN (665.97): C 68.53, H 6.66, N 2.10; found: C 68.34, H 6.80, N 1.95.

### Crystallographic methods

Crystals were mounted on MicroMounts (MiTeGen) by using YR-800 perfluoropolyether oil and cooled rapidly to 90 or 120 K in a stream of cold nitrogen using an Oxford Cryosystems low-temperature device.[31] Data for **4** were collected on an Oxford Diffraction SuperNova diffractometer, equipped with a mirror-monochromated Cu_Kα_ source (*λ*=1.5418 Å). Data for compounds **2** and **6** were collected on Oxford Diffraction GV1000 diffractometers, equipped with a mirror-monochromated Cu_Kα_ source (*λ*=1.5418 Å). Programs used were CrysAlisPro,[32] SHELXS,[33] SHELXL[33] and OLEX2[34] (structure solution, structure refinement and molecular graphics).

In compound **2**, *tert*-butyl groups C(29)–C(32) and C(33)–C(36) are rotationally disordered. The occupancy of the disorder components was refined competitively, converging at ratios of 0.634(6):0.366(6) and 0.56(2):0.44(2), respectively. Within the disordered methyl groups 1,2-C–C distances were restrained to be approximately equal, as were 1,3-C–C distances. Enhanced rigid-bond restraints were applied to the whole structure. In compound **6**, positional disorder was identified for atom Mg(2). This was modelled over two positions and the occupancies of the two components refined competitively, converging at a ratio of 0.66(7):0.34(7). Chemically equivalent bonds to the disordered magnesium atoms were restrained to be approximately equal. The *tert*-butyl group C(74)–C(76) is disordered over two orientations. The occupancies of the two components were refined competitively, converging at a ratio of 0.785(14):0.215(14). Within the disordered methyl groups 1,2-C–C distances were restrained to be approximately equal, as were 1,3-C–C distances. Finally, minor component atom C(75 a) was refined isotropically, whereas enhanced rigid-bond restraints were applied to the rest of the *tert*-butyl group, and to the hexane solvent molecule.

### Crystal data for 2

C_80_H_96_K_2_N_2_O_2_**⋅**C_6_H_14_, *M*_r_=1281.95 g mol^−1^, 0.12×0.12×0.15 mm^3^, *T*=120(2) K, monoclinic, space group *P*2_1_/*n*, *a*=11.0990(5), *b*=20.8684(9), *c*=17.8531(7) Å, *β*=97.589(4)°, *V*=4098.9(3) Å^3^, *Z*=2, *ρ*_calcd_=1.039 g cm^−3^, *μ*=1.345 mm^−1^, *F*(000)=1388. A total of 17 426 reflections were measured, of which 8126 were unique, with *R*_int_=0.044. Final *R*_1_ (*wR*_2_)=0.0819 (0.2234) with GOF=1.08. Minimal and maximal residual electron densities are −0.25 and 0.84 e Å^3^, respectively.

### Crystal data for 4

C_36_H_56_IMgNO_2_, *M*_r_=686.02 g mol^−1^, 0.13×0.15×0.18 mm^3^, *T*=90(2) K, orthorhombic, space group *P*2_1_2_1_2_1_, *a*=9.3083(3), *b*=12.9098(3), *c*=29.3270(7) Å, *V*=3524.19(16) Å^3^, *Z*=4, *ρ*_calcd_=1.293 g cm^−3^, *μ*=7.532 mm^−1^, *F*(000)=1440. A total of 10 404 reflections were measured, of which 6035 were unique, with *R*_int_=0.047. Final *R*_1_ (*wR*_2_)=0.0394 (0.0932) with GOF=1.03. Flack parameter=0.022(6).[35, 36] Minimal and maximal residual electron densities are −0.71 and 0.70 e Å^3^, respectively.

### Crystal data for 6

C_42_H_52_IMgNO**⋅**0.5(C_6_H_14_), *M*_r_=781.14 g mol^−1^, 0.07×0.07×0.28 mm^3^, *T*=120(2) K, triclinic, space group *P*$\bar 1$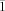, *a*=12.3913(10), *b*=15.8801(8), *c*=21.9933(10) Å, *α*=101.923(4), *β*=96.001(5), *γ*=92.320(5)°, *V*=4202.6(5) Å^3^, *Z*=4, *ρ*_calcd_=1.235 g cm^−3^, *μ*=6.365 mm^−1^, *F*(000)=1636. A total of 33 014 reflections were measured, of which 16 670 were unique, with *R*_int_=0.102. Final *R*_1_ (*wR*_2_)=0.0786 (0.2245) with GOF=1.03. Minimal and maximal residual electron densities are −1.65 and 1.45 e Å^3^, respectively.

### Percent buried volume calculations

Percent buried volume calculations were performed using the web application Samb*V*ca.[37] XYZ files derived from the crystallographic data were used as input files, with the coordinated THF molecules (**1**, **2**, **4** and **6**) and iodine atoms (**4** and **6**) removed; additionally, in the atoms list the metals were renamed to hydrogen. The calculations were run by using Bondi radii scaled by 1.17 for the atoms, mesh spacing *s*=0.05 Å and sphere of *R*=3.5 Å.[37, 38] The centre of the sphere was positioned at 2.10 Å from the nitrogen donor, coplanar to the pyrrolyl ring and equidistant with respect to the *α*-carbons.

CCDC-1039042 http://www.ccdc.cam.ac.uk/cgi-bin/catreq.cgi(**2**), CCDC-1039043 http://www.ccdc.cam.ac.uk/cgi-bin/catreq.cgi(**4**), CCDC-1039044 http://www.ccdc.cam.ac.uk/cgi-bin/catreq.cgi(**6**), CCDC-1039045 http://www.ccdc.cam.ac.uk/cgi-bin/catreq.cgi(**L^3^**H polymorph 1) and CCDC-1039046 http://www.ccdc.cam.ac.uk/cgi-bin/catreq.cgi(**L^3^**H polymorph 2) contain the supplementary crystallographic data for this paper. These data can be obtained free of charge from The Cambridge Crystallographic Data Centre via http://www.ccdc.cam.ac.uk/data_ request/cif.

## Supporting Information

As a service to our authors and readers, this journal provides supporting information supplied by the authors. Such materials are peer reviewed and may be re-organized for online delivery, but are not copy-edited or typeset. Technical support issues arising from supporting information (other than missing files) should be addressed to the authors.

miscellaneous_information
