# Supplementary material for: Tuning Coordination in s-Block Carbazol-9-yl Complexes
Source: Chemistry. 2015 Mar 17;21(18):6949–56. doi: 10.1002/chem.201406490 (PMC4641456; doi:10.1002/chem.201406490)
Supplement: Supplementary file 1 — miscellaneous_information [file chem0021-6949-sd1.pdf]

CHEMISTRY
A European Journal

Supporting Information

# Tuning Coordination in s-Block Carbazol-9-yl Complexes

Fabrizio Ortu,[a, b] Graeme J. Moxey,[a, c] Alexander J. Blake,[a] William Lewis,[a] and Deborah L. Kays*[a]

chem_201406490_sm_miscellaneous_information.pdf

# Supporting Information

## Contents

Page S2: Synthesis and characterization of carbazoles $\mathbf{L}^2$H and $\mathbf{L}^3$H.

Page S3: Crystal Structure of $\mathbf{L}^3$H.

Page S4: Spacefilling diagram of complexes **4** and **6a**.

Page S5: References.

**Synthesis and characterization of carbazoles $L^2H$ and $L^3H$**

Diaryl-substituted carbazoles, along with starting materials for the Suzuki coupling reactions, $ArB(OH)_2$ [Ar = Xyl (Xyl = 3,5-$Me_2C_6H_3$), Mes (Mes = 2,4,6-$Me_3C_6H_2$)] and $^tBu_2Br_2$CarbH, were synthesized by literature methods.[S1-S3] All other reagents were used as supplied. Solutions of the chosen arylboronic acid in ethanol (400 $cm^3$) and $Na_2CO_3$ (1M, 200 $cm^3$) were added to a solution of $^tBu_2Br_2$CarbH in toluene (600 $cm^3$), with a carbazole/$ArB(OH)_2$ molar ratio of approximately 1:10; the mixture was purged with argon over a period of 30 minutes. A solution of $[Pd(PPh_3)_4]$ (7% mol/mol) in toluene (200 $cm^3$) was added and the mixture heated at 80 °C for 72 hours. The reaction was filtered while hot and the organic and aqueous layers separated. The organic layer was washed with brine (2 × 100 $cm^3$) and dried over $MgSO_4$. The remaining organic solution was concentrated and triturated with ethanol, affording $^tBu_2Ar_2$CarbH as an off-white solid.

**1,8-Xyl$_2$-3,6-$^t$Bu$_2$carbH ($L^2H$):** From 2.0 g of $^tBu_2Br_2$carbH (4.5 mmol) and 6.50 g of $XylB(OH)_2$ (0.04 mol); 1.52 g of $\mathbf{L}^2$H (3.1 mmol, yield 69%). $^1$H NMR ($CDCl_3$, 298 K, 400.07 MHz): δ = 1.53 (s, 18H, C(C$H_3$)$_3$), 2.45 (s, 12H, Ar-C$H_3$), 7.05 (br, 2H, Ar-C*H*), 7.35 (br, 4H, Ar-C*H*), 7.50 (br, 2H, carb-C$H^{2,7}$), 8.10 (br, 2H, carb-C$H^{4,5}$), 8.40 (br, 1H, N*H*) ppm. $^{13}$C{$^1$H} NMR ($CDCl_3$, 298 K, 100.63 MHz): δ = 21.5 (Ar-*C*$H_3$), 32.1 (C(*C*$H_3$)$_3$), 34.8 (*C*($CH_3$)$_3$), 115.4 (carb-*C*$H^{4,5}$), 123.5 (carb-*C*$H^{2,7}$), 123.9 (carb-$C^{4a,4b}$), 124.2 (carb-$C^{8a,9a}$), 126.0 (Ar-*C*H), 129.0 (Ar-*C*H), 135.8 (carb-$C^{1,8}$), 138.7 (Ar-*C*($CH_3$)), 139.6 (Ar-*C*), 142.8 (carb-$C^{3,6}$) ppm. IR: ν = 3465 (md), 1600 (st), 1287 (md), 1261 (md), 1230 (md), 1024 (br md), 870 (wk), 859 (wk), 851 (md), 801 (wk), 709 (md), 662 (md), 650 (md), 596 (st), 545 (wk), 522 (wk), 522 (wk), 434 (md) $cm^{-1}$.

**1,8-Mes$_2$-3,6-$^t$Bu$_2$carbH ($L^3H$):** From 2.0 g of $^tBu_2Br_2$carbH (4.5 mmol) and 8.2 g of $MesB(OH)_2$ (0.05 mol); 1.57 g of $\mathbf{L}^3$H (3.1 mmol, yield 68%). $^1$H NMR ($CDCl_3$, 298 K, 400.07 MHz): δ = 1.48 (s, 18H, C(C$H_3$)$_3$), 1.98 (s, 12H, Ar-C$H_3$), 2.37 (s, 6H, Ar-C$H_3$), 7.00 (br, 4H, Ar-C*H*), 7.21 (br, 1H, N*H*), 7.22 (d, $^4J_{HH}$ = 1.9 Hz, 2H, carb-C$H^{2,7}$), 8.14 (d, $^4J_{HH}$ = 1.9 Hz, 2H, carb-C$H^{4,5}$) ppm. $^{13}$C{$^1$H} NMR ($CDCl_3$, 298 K, 100.63 MHz): δ = 20.2 (Ar-*C*$H_3$), 21.0 (Ar-*C*$H_3$), 32.0 (C(*C*$H_3$)$_3$), 34.8 (*C*($CH_3$)$_3$), 114.6 (carb-*C*$H^{4,5}$), 122.8 (carb-$C^{4a,4b}$), 123.3 (carb-$C^{8a,9a}$), 125.0 (carb-*C*$H^{2,7}$), 128.5 (Ar-*C*H), 135.1 (Ar-*C*), 136.2 (carb-$C^{1,8}$), 137.1 (Ar-*C*($CH_3$)), 137.2 (Ar-*C*($CH_3$)), 142.7 (carb-$C^{3,6}$) ppm. IR: ν = 3458 (md), 1609 (md), 1260 (br md), 1097 (wk), 1048 (br md), 870 (md), 845 (wk), 803 (md), 675 (wk), 651 (wk), 625 (wk), 605 (wk), 557 (md) $cm^{-1}$.

**Crystal Structure of L$^3$H**

Crystals were mounted on MicroMounts™ (MiTeGen) using YR-800 perfluoropolyether oil and cooled rapidly to 120 K in a stream of cold nitrogen using an Oxford Cryosystems low-temperature device.[S4] Data for **L**$^3$H were collected on and Oxford Diffraction GV1000 diffractometer, equipped with a mirror-monochromated Cu *K*α source (λ = 1.5418 Å). Programs used were CrysAlisPro,[S5] SHELXS,[S6] SHELXL[S6] and OLEX2[S7] (structure solution, structure refinement and molecular graphics). Two different polymorphs of **L**$^3$H crystals were obtained. In the first polymorph of **L**$^3$H positional disorder was identified for the methyl carbon atoms C(32), C(33) and C(34): occupancies of the two components were refined competitively converging at a ratio of 0.696(10):0.304(10). Another polymorph of **L**$^3$H (**2**, below) was also obtained, and also in this case positional disorder was identified for one of the *tert*-butyl fragments, C(36)-C(38). This was modelled over two positions and the two components were refined competitively converging at a ratio of 0.55(1):0.45(1). Data for both are given below:

*Crystal data for **L**$^3$H:* $C_{38}H_{45}N$, $M_r$ = 515.75, 0.07 x 0.12 x 0.15 mm$^3$, T = 120(2) K, monoclinic, space group $P2_1/c$, $a$ = 9.14410(14) Å, $b$ = 31.7460(5) Å, $c$ = 10.93464(16) Å, $\beta$ = 102.9120(14)°, $V$ = 3093.94(8) Å$^3$, $Z$ = 8, $D_{calcd}$ = 1.107 g cm$^{-3}$, $\mu$ = 0.468 mm$^{-1}$, $F$(000) = 1120. A total of 12410 reflections were measured, of which 6176 were unique, with $R_{int}$ = 0.024. Final $R_1$ ($wR_2$) = 0.0437 (0.121) with GOF = 1.02. Min. and max. residual electron densities –0.20 and 0.22 e/Å$^3$.

*Crystal data for **L**$^3$H (2):* $C_{38}H_{45}N$, $M_r$ = 515.75, 0.12 x 0.23 x 0.29 mm$^3$, T = 120(2) K, monoclinic, space group *Cc*, $a$ = 21.8643(3) Å, $b$ = 14.01221(14) Å, $c$ = 10.90010(13) Å, $\beta$ = 110.2710(15)°, $V$ = 3132.61(7) Å$^3$, $Z$ = 4, $D_{calcd}$ = 1.094 g cm$^{-3}$, $\mu$ = 0.462 mm, $F$(000) = 1120. A total of 9722 reflections were measured, of which 5205 were unique, with $R_{int}$ = 0.009. Final $R_1$ ($wR_2$) = 0.0276 (0.0745) with GOF = 1.04. Min. and max. residual electron densities –0.15 and 0.17 e/Å$^3$.

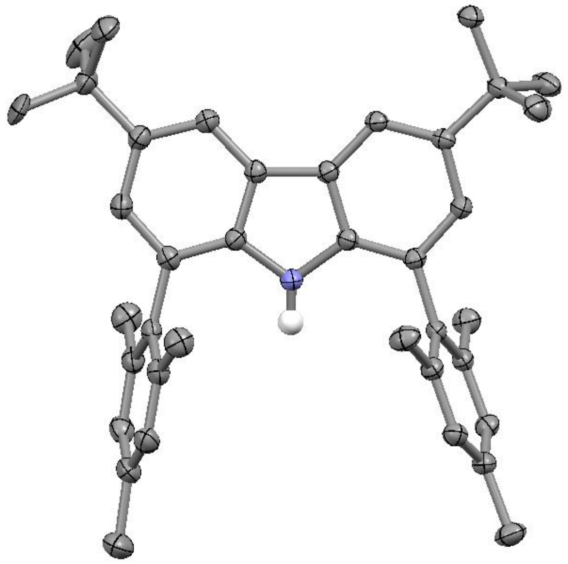

**Figure S1.** Molecular structure of **L**$^{3}$H, with displacement ellipsoids set at 50% probability level. Hydrogen atoms, with the exception of the NH hydrogen, have been omitted for clarity. In the solid state, the mesityl substituents are almost perpendicular to the carbazole plane.

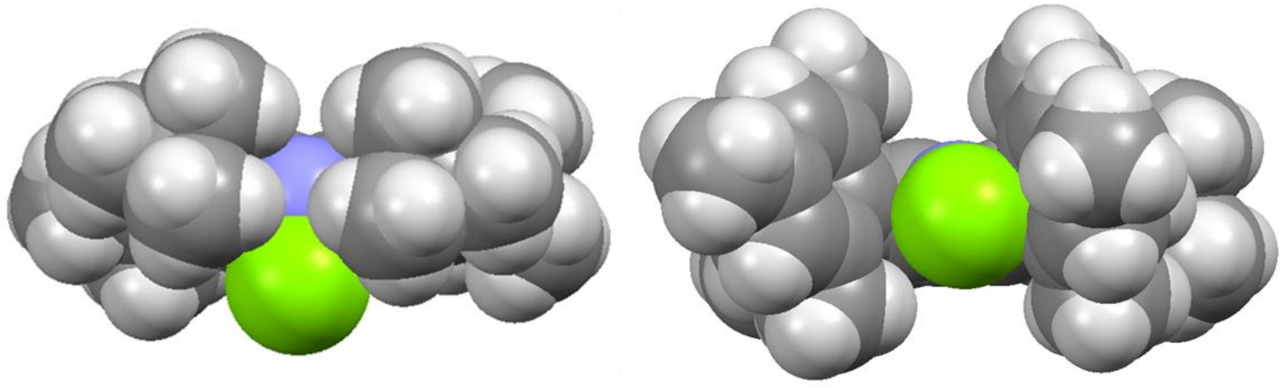

4

6a

**Figure S2.** Space-filling diagram representing **4** (left) and **6a** (right). Coordinated iodide anions and THF molecules omitted for clarity. Atoms represented: carbon (gray), hydrogen (light gray), magnesium (green), nitrogen (blue).

**References**

S1. Spitzmesser, S. K.; Gibson, V. C. *J. Organomet. Chem.* **2003**, *673*, 95.

S2. Coombs, N. D.; Stasch, A.; Cowley, A.; Thompson, A. L.; Aldridge, S. *Dalton Trans.* **2008**, 332.

S3. Britovsek, G. J. P.; Gibson, V. C.; Hoarau, O. D.; Spitzmesser, S. K.; White, A. J. P.; Williams, D. J. *Inorg. Chem.* **2003**, *42*, 3454.

S4. Cosier, J.; Glazer, A. M. *J. Appl. Crystallogr.* **1986**, *19*, 105.

S5. CrysAlisPro, Version 1.171.33.55, Oxford Diffraction Ltd; Clark, R. C.; Reid, J. S. *Acta Crystallogr., Sect A: Fundam. Crystallogr.* **1995**, *51*, 887.

S6. Sheldrick, G. M. *Acta Crystallogr., Sect. A: Fundam. Crystallogr.* **2008**, *64*, 112.

S7. Dolomanov, O. V.; Bourhis, L. J.; Gildea, R. J.; Howard, J. A. K.; Puschmann, H. *J. Appl. Crystallogr.* **2009**, *42*, 339.
